# Toward a Unified Theory of Customer Continuance Model for Financial Technology Chatbots

**DOI:** 10.3390/s21175687

**Published:** 2021-08-24

**Authors:** Stanley Y. B. Huang, Chih-Jen Lee, Shih-Chin Lee

**Affiliations:** 1 Master Program of Financial Technology, School of Financial Technology, Ming Chuan University, Taipei 111, Taiwan; yanbin@mail.mcu.edu.tw (S.Y.B.H.); louis@mail.mcu.edu.tw (C.-J.L.); 2 Department of Finance, Chihlee University of Technology, New Taipei 220, Taiwan

**Keywords:** financial technology chatbots, continuance intention, latent growth curve modeling, technology acceptance model

## Abstract

With the popularity of financial technology (fintech) chatbots equipped with artificial intelligence, understanding the user’s response mechanism can help bankers formulate precise marketing strategies, which is a crucial issue in the social science field. Nevertheless, the user’s response mechanism towards financial technology chatbots has been relatively under-investigated. To fill these literature gaps, latent growth curve modeling was adopted by the present research to survey Taiwanese users of fintech chatbots. The present study proposed a customer continuance model to predict continuance intention for fintech chatbots and that cognitive and emotional dimensions positively influence the growth in a user’s attitude toward fintech chatbots, which in turn, positively influences continuance intention over time. In total, 401 customers of fintech chatbots were surveyed through three time points to examine the relationship between these variables over six months. The results support the theoretical model of this research and can advance the literature of fintech chatbots and the information technology adoption model.

## 1. Introduction

To reach sustainable development, bankers should make a strategy to attract more customers [1] and to gain commercial benefits [2]. Financial technology (fintech) services, such as fintech chatbots, have been confirmed as innovative services to improve customers’ experiences [3]. The fintech chatbot is an online service that responds to consumers by an artificial intelligence algorithm [4,5]. However, the vigorous growth of fintech investment [6] does not fit the fintech use growth because the psychological mechanisms behind why people use fintech has been relatively less investigated. Previous studies have found that the driving factors of continuous intention in online banking services are based on utilitarian orientation, and few studies have focused on emotional orientation [7]. Indeed, the literature on adopting intention in the information system environment is almost based on the traditional information technology adoption model [8,9,10]. Therefore, it is important to incorporate the emotional dimension into traditional adoption models to open the black box of customers’ behavior mechanisms.

As discussed above, previous studies of the information technology adoption model have studied continuance intention and its antecedents almost based on utilitarian orientation [11,12,13]. Therefore, we propose the customer continuance model to describe how ease of use, usefulness, pleasure, and arousal lead to a stronger change in attitudes toward fintech chatbots, which, in turn, leads to a positive change in the continuance intention. The customer continuance model combines the technology acceptance model (TAM) (utilitarian orientation) with Russell’s [14] pleasure and arousal (emotional orientation) to predict the continuance intention for fintech chatbots. In addition, this research employs a non-random empirical survey method [15] instead of a random experimental design [16], because this research studies 401 Taiwanese users of fintech chatbots in three stages over a six month period through latent growth curve modeling (LGCM) [17,18,19] to explore whether adding emotional factors to the TAM can better predict continuous intention.

In sum, this research adopts a longitudinal survey because perceived usefulness, perceived pleasure, perceived arousal, attitudes toward information technology, and continuance intention are all regarded as trait variables (e.g., persistent personality variables) [20,21,22]. Additionally, the previous study on continuance intention was almost just cross-sectional samples [23,24,25], so there is currently little evidence of the causal relationship between continuance intention and its antecedent. By using the LGCM, this research can fill these literature gaps in the information technology field.

## 2. Hypothesis Development and Literature Review

### Russell’s Affect Model and the TAM

The TAM believes that if ease of use and usefulness (cognition dimension) can arouse individual attitudes about technology, then it can affect use intention [10]. Previous studies have confirmed this hypothesis to indicate the importance of the emotional factor in determining customer attitudes [26,27,28], such as hedonic feelings of satisfaction and enjoyment. This study retrieved relevant emotional literature and found that Russell’s [14] emotional model can represent almost all emotional responses, such as satisfaction, enjoyment, happiness, and excitement. Therefore, this research uses perceived pleasure and perceived arousal based on Russell [14] to express emotional orientation and integrate these two emotional factors in the TAM to propose the customer continuance model.

## 3. Evaluation Procedure

### 3.1. Research Model and Hypotheses

**Figure 1:**
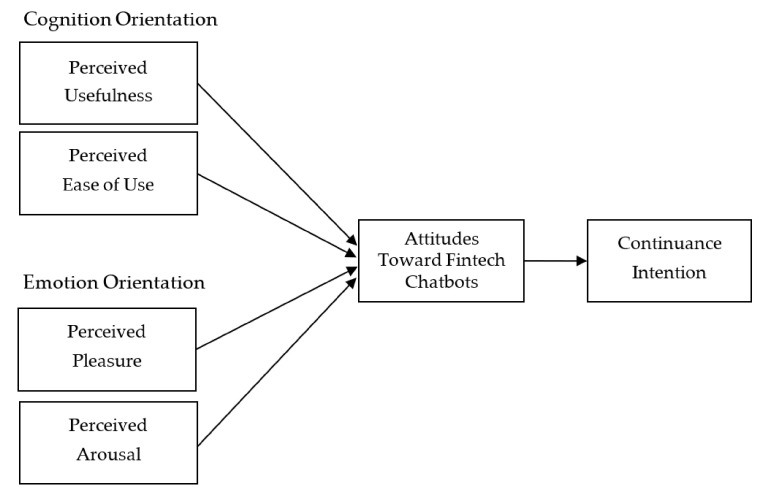
Proposed model.

The present study proposes the customer continuance model to supplement the original TAM, which contains utilitarian dimensions (ease of use and usefulness), two emotional dimensions (pleasure and arousal), the customer’s attitudes toward fintech chatbots, and the customer’s continuance intention (please see Figure 1).

Usefulness refers to individuals having faith in information technology being able to increase productivity [10]. In a customer setting, usefulness is described by the present study as the extent to which customers have faith that the fintech chatbot can provide interest in performing specific tasks. This is also a predecessor of attitudes toward technology based on Davis and his colleagues’ model [10]. Many studies have found the impact of perceived usefulness on attitudes toward new technology services [29,30]. Therefore:

**Hypothesis** **1.** *Greater degree of usefulness in the first phase of time will lead to a significantly stronger change in attitudes toward fintech chatbots.*

Ease of use refers to individuals having faith in the simplification of information technology [10]. In a customer setting, ease of use is described as customers having faith in a fintech chatbot that is free of effort. This is also a significant antecedent of attitudes toward information technology according to the original TAM [10]. Past researchers have surveyed the relationship between attitudes toward new technology services and ease of use [31,32], and have adopted ease of use as a significant driving antecedent in predicting use attitudes toward fintech [3,33]. Therefore:

**Hypothesis** **2.** *Greater degree of ease of use in the first phase of time will lead to a significantly stronger change in attitudes toward fintech chatbots.*

Pleasure means the extent to which individuals go through a cheerful response to certain stimuli [14]. In a customer setting, perceived pleasure denotes the level to which customers go through a cheerful response to the stimuli of fintech chatbots. Arousal means the extent to which individuals’ excited states respond to certain stimuli [14]. In a customer setting, perceived arousal denotes the level to which customers’ excited states respond to the stimuli of fintech chatbots. Past research has examined the emotional factors as antecedents of attitudes toward new technology services [34,35,36]. Past studies have also found that perceived pleasure and perceived arousal influence attitudes toward the adoption of digital devices, commercial sites, and hedonic information systems [37,38,39]. Therefore:

**Hypothesis** **3.** *Greater degree of pleasure in the first phase of time will lead to a significantly stronger change in attitudes toward fintech chatbots.*

**Hypothesis** **4.** *Greater degree of arousal in the first phase of time will lead to a significantly stronger change in attitudes toward fintech chatbots.*

In the original TAM, attitudes toward information technology means the assessment and judgment of the user intention for information technology [10], which is an outcome of cognitive factors (ease of use and usefulness). However, the attitudes toward information technology may be confusing because the empirical studies on attitudes toward information technology have multiple assumptions. For example, some empirical studies removed attitudes from the TAM [40,41,42], and other studies have expanded the attitude effect on continuance intention [43,44] and purchase intention [45,46,47]. In summary, it is crucial to clarify the conditions under which attitude factors can link cognition, emotion, and intention.

The present study proposes that attitudes toward fintech chatbots should be linked to cognition factors (usefulness and ease of use) and emotion (pleasure and arousal) because the attitudes in an information technology setting should include not only cognition-based factors but also emotion-based factors. Indeed, previous studies have confirmed this hypothesis, and attitudes toward information technology have been examined as an intermediary between emotion–intention links [26,27,28]. In addition, attitudes toward information technology have also been examined as a mediator between cognition–intention links in the information technology adoption model [8,9,10]. These findings are reasonable because attitudes toward information technology have been theorized as an intermediary between cognition, emotion, and intention links for a long time [48].

Although attitudes toward information technology are connected to information technology adoption intention, many recent studies have suggested that attitudes toward information technology are an antecedent of continuance intention [43,44]. Indeed, an individual’s attitude toward a certain object can be fostered by their individual experience, which is similar to a continuing desire, preference, and identity, and is likely to form loyalty [49,50]. In other words, individuals’ attitudes toward information technology can affect individuals’ continuance intention (loyalty). Therefore:

**Hypothesis** **5.** *More positive growth in attitudes toward fintech chatbots will lead to a significantly stronger change in continuance intention.*

### 3.2. Method

#### 3.2.1. Research Population

This research proposes the customer continuance model to describe how the usefulness, ease of use, pleasure, and arousal in the first phase of time affects the change in attitudes toward fintech chatbots, and then affects the growth in continuance intention. The proposed model is different from the traditional information technology model and continuously advances the literature of information technology use intention.

To confirm the validity, reliability, and causal relationship of the customer continuance model, the present study gathered samples at three time points over six months. The LGCM was employed to analyze the causal relationships of these variables [17].

To collect the subjects who had used fintech chatbots in the past three months (selection criteria), we recruited a suitable sample and obtained a list of 500 users who met this selection criteria. This research sent 500 emails to subjects to confirm their willingness to participate in the investigation and provided a gift certificate worth USD four. In total, 500 customers responded to the email and participated in the survey.

These customers’ evaluations of the usefulness, ease of use, pleasure, arousal, attitudes toward fintech chatbots, and continuance intention were obtained at the first time point; this research asked these customers again after three months (the second time point) about their attitudes toward fintech chatbots and their continuance intention. This research again examined the attitudes toward fintech chatbots and continuance intention six months later (the third time point). The three-month survey interval was used because growth in behavioral intention should be visible within the three-month interval [51,52,53,54]. The final sample of this research was 401 customers. The demographics are as Table 1.

#### 3.2.2. Research Instruments

To confirm the translation quality between Chinese and English, the present study referred to Reynolds and his colleagues’ [55] work. Besides, the present study used a self-report questionnaire with a seven-point Likert scale to measure these variables.

This research used the scales developed by the previous study because these had confirmed their reliability and validity. Ease of use and usefulness were assessed by Lund’s [56] scale. Pleasure and arousal were assessed by Moore and Benbasat’s [57] scale. Attitudes toward fintech chatbots were assessed by Chen and Wells’ [58] scale. Continuance intention was measured by Bhattacherjee’s [59] scale.

## 4. Analysis Results and Discussion

### 4.1. Model Validation

The reliability and validity were tested by the analysis technique of confirmatory factor (CA). Next, we adopted LGCM to test the proposed model.

#### Model Measurement

This research employed CA to test the model’s variables (please see Table 2). The average variation extraction of each variable in the theoretical model of the present study was higher than 0.66 and the composite reliability of these variables were all greater than 0.76. The model fit for RMSEA and RMR was less than 0.08 and 0.05, respectively. The model fit for NFI, GFI, and CFI was higher than 0.9, respectively.

### 4.2. Hypotheses Testing and Analysis Results

**Figure 2:**
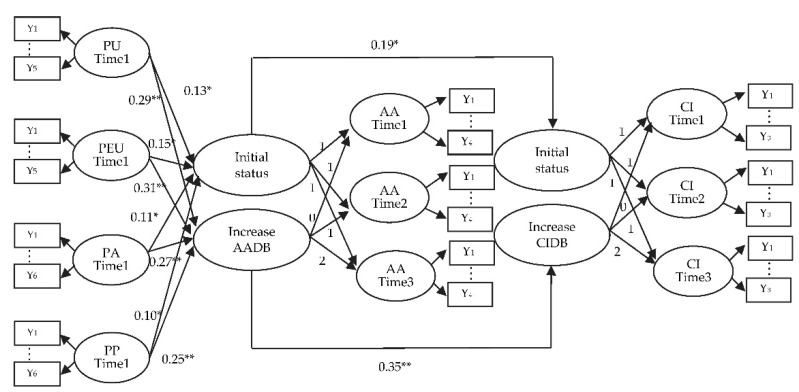
The LGCM. Note: PU = Perceived Usefulness; PEU= Perceived Ease of Use; PA = Perceived Arousal; PP = Perceived Pleasure; AA = Attitudes toward Fintech Chatbots; CI = Continuance Intention; AADB = Attitudes toward Fintech Chatbots development behavior; CIDB = Continuance Intention development behavior. Yn = Measurement items. Exogenous Variables: PU, PEU, PA, and PP; Endogenous Variables: AADB, and CIDB. * *p* < 0.05; ** *p* < 0.01.

The LGCM [17] has been gradually applied to interdisciplinary fields [60,61,62] because of its powerful ability to capture the change of variables over time. The analysis results are shown in Table 3. The ease of use (β = 0.31, *p* < 0.01), usefulness (β = 0.29, *p* < 0.01), pleasure (β = 0.27, *p* < 0.01), and arousal (β = 0.25, *p* < 0.01) in the first phase of time significantly influenced the growth in attitudes toward fintech chatbots (please see Figure 2). Hypothesis 1, 2, 3, and 4 propose that if a customer perceived more usefulness, ease of use, pleasure, and arousal in the first phase of time when using the fintech chatbot, then this may influence his or her change in attitude toward fintech chatbots, which is supported. That is, the customer who perceived more ease of use, usefulness, pleasure, and arousal at the first time point may have improved their attitude toward fintech chatbots over time.

Improved growth in attitudes toward fintech chatbots (β = 0.35, *p* < 0.01) significantly influenced growth in continuance intention (please see Figure 2). Hypothesis 5 proposes that if a customer has increased growth in attitudes toward fintech chatbots, then this may cause improved growth in their continuance intention, which is supported.

The statistical explanatory power of the customer continuance model is 54% for the behavior intention, and the explanatory power of the original TAM is 34% for the behavior intention [10], which has a 58.8% improvement rate of statistical explanatory power. In addition, we show the explanatory power of other information technology adoption models as in Table 4, and the explanatory power of the customer continuance model for the behavior intention is greater than these models.

Besides, the present study employed a chi-squared different test to compare the value of chi-squared between the customer continuance model and the original TAM, and results showed that the value of chi-squared for TAM [10] was worse than the customer continuance model (∆ chi-squared is 103 (*p* < 0.01)), which was a significant difference. Given these results, the customer continuance model should improve upon the original TAM.

### 4.3. Findings and Discussion

This research is the first to employ LGCM and longitudinal data over six months to conceptualize the customer continuance model, and demonstrates that usefulness, ease of use, pleasure, and arousal affects the growth in attitudes toward fintech chatbots. This, then, significantly affects the growth in continuance intention, which proves the high level of validity and reliability.

Next, the customer continuance model includes not only emotional factors but also cognitive factors, so the statistical explanatory power of the customer continuance model reaches 54%, which is better than previous studies on the TAM with a statistical explanatory power between 17% and 33% [10,70]. Indeed, the factors that drive the customer’s attitudes towards fintech chatbots may not only be cognitive factors, because the formation of customer attitudes is a complex process. Finally, the empirical results offer contributions for information technology behavior models and practices. Previous studies have almost totally neglected emotional factors to predict behavior intentions [71,72]. By including emotional factors in the model, the customer continuance model can significantly improve the predictive power for continuance intention. Thus, it is important that the banks keep in mind that cognitive factors and emotional factors can both affect the customer’s attitudes and continuance intention. Therefore, the bank’s owners should not only improve the usefulness and ease of use of fintech chatbots but also design chatbots that can increase the customer’s pleasure and arousal. Besides, the vendors can employ marketing communication to increase cognition-based and emotional factors. For example, the vendors can use advertising with customized campaigns to demonstrate the usefulness and ease of use of fintech chatbots, and the pleasure and arousal they can provide, in order to affect customer perception.

## 5. Conclusions and Future Work

The present study incorporates emotional factors into the original TAM to improve the ability of TAM to predict continuance intention and proposes the customer continuance model, which opens the black box of customer psychology when using fintech chatbots. This research demonstrates that the customer continuance model has a better statistical explanatory power than the original TAM and is the first to establish a milestone to propose the novel customer continuance model through the LGCM to predict continuance intention. The results can advance the literature on the information technology adoption model and can also provide bankers with support to improve continuance intention, thereby transforming customers’ continuance intention into buying behaviors.

Although the present research combines emotional factors and cognition-based factors to propose the customer continuance model, different emotional and cognitive factors may be crucial in different contexts. Future research should identify the other emotions and cognitions involved in different contexts because the customer continuance model may lose some emotion- and cognition-based factors in different regions/countries. Next, this research only puts forward the emotion- and cognition-based factors for the customer continuance model to meet simplicity. Future research should explore other important antecedents that could be included in the customer continuance model to supplement the completeness of the model. Finally, the LGCM and longitudinal data were employed to analyze the customer continuance model’s reliability and validity, but the longitudinal data over six months may not be enough to make causal inferences. Future research should verify the causality of the customer continuance model by using more samples and longer-term longitudinal data.

## Figures and Tables

**Table 1:** Demographics of the samples (Sample size = 401).

Measurement	Item	
Gender	Female	40%
	Male	60%
Use tenure	More than one year	40%
	Less than one year	60%
Education	College education or above	89%
	Less than a college education	11%
Frequency of using chatbots	Once everyone week	30%
	Once every two weeks	60%
	Once every three weeks or above	30%

**Table 2:** The Analysis of Validity and Reliability.

Construct	Item	Factor Loading	Composite Reliability	Average Variance Extracted
Usefulness	U1	0.82	0.90	0.66
	U2	0.79		
	U3	0.81		
	U4	0.83		
	U5	0.81		
Ease of use	E1	0.81	0.90	0.66
	E2	0.83		
	E3	0.81		
	E4	0.83		
	E5	0.79		
Pleasure	P1	0.83	0.91	0.66
	P2	0.82		
	P3	0.81		
	P4	0.79		
	P5	0.79		
	P6	0.82		
Arousal	A1	0.79	0.92	0.66
	A2	0.82		
	A3	0.81		
	A4	0.83		
	A5	0.84		
	A6	0.82		
Attitudes toward fintech chatbots	AA1	0.83	0.89	0.67
	AA2	0.80		
	AA3	0.82		
	AA4	0.83		
Continuance intention	C1	0.82	0.86	0.67
	C2	0.81		
	C3	0.83		

**Table 3:** Analysis results.

Hypothesis		Analysis Results
H_1_	Ease of use (phase 1 time) →Attitudes toward fintech chatbots	0.31 **
H_2_	Usefulness (phase 1 time) →Attitudes toward fintech chatbots	0.29 **
H_3_	Pleasure (phase 1 time) →Attitudes toward fintech chatbots	0.27 **
H_4_	Arousal (phase 1 time) →Attitudes toward fintech chatbots	0.25 **
H_5_	Attitudes toward fintech chatbots →Continuance intention	0.35 **

Note: ** *p* < 0.01.

**Table 4:** The statistical explanatory power for the information technology adoption models.

Empirical Model	Explanatory Power	References
TAM	34%	[10]
TAM	37%	[63]
TPB	37%	[64]
TPB	41%	[65]
TRA	20%	[66]
TRA	19%	[67]
UTAUT	31%	[68]
UTAUT2	44%	[69]

Note: TAM = Technology Acceptance Model; TPB = Theory of Planned Behavior; TRA = Theory of Reasoned Action; UTAUT = Unified Theory of Acceptance and Use of Technology; UTAUT2 = Unified Theory of Acceptance and Use of Technology 2.

## Data Availability

Not applicable.
